# Exploration of chemical compositions in different germplasm wolfberry using UPLC-MS/MS and evaluation of the *in vitro* anti-inflammatory activity of quercetin

**DOI:** 10.3389/fphar.2024.1426944

**Published:** 2024-07-04

**Authors:** Tian Lan, Guozhen Duan, Youchao Qi, Maged Almezgagi, Guanghui Fan, Yonggui Ma

**Affiliations:** ^1^ College of Agriculture and Animal Husbandry, Qinghai University, Xining, China; ^2^ College of Medical, Qinghai University, Xining, China; ^3^ College of Tibetan Medicine, Qinghai University, Xining, China; ^4^ College of Agriculture and Forestry, Qinghai University, Xining, China; ^5^ High-altitude Medical Research Center, the Key Laboratory of High-altitude Medical Application of Qinghai Province, Department of Immunology, Medical College of Qinghai University, Xining, China; ^6^ Key Laboratory of Medicinal Animal and Plant Resources of Qinghai Tibetan Plateau, Qinghai Normal University, Xining, China

**Keywords:** wolfberry, metabolomics, flavonoids, HUVEC cell, quercetin, anti-inflammation

## Abstract

Wolfberry, esteemed as a traditional Chinese medicinal material and functional food, is replete with nutrients and boasts a diverse array of health benefits, including hypoglycemic, antitumor, antioxidant, anti-inflammatory, and immune-enhancing properties. Notably, inflammation is a pivotal factor in the onset and progression of numerous diseases. Despite this, there is a paucity of research on the comprehensive evaluation of the components found in different wolfberries, and the exploration of their primary active components is limited. To address this issue, we conducted a comprehensive targeted metabolomics analysis, employing statistical methods such as principal component analysis (PCA), orthogonal partial least squares discriminant analysis (OPLS-DA), KEGG pathway analysis, and volcano plots to delineate the compositional differences among red, black, and yellow wolfberries. Furthermore, we investigated the anti-inflammatory effects of their primary components through *in vitro* experiments. Our analysis revealed a total of 1,104 chemical compositions in the three wolfberries, with alkaloids, phenolic acids, flavonoids, and lipids being the predominant nutritional components. KEGG enrichment analysis indicated that these compositions were primarily involved in the biosynthesis of secondary metabolites, ABC transport, and galactose metabolism pathway. Moreover, our study demonstrated that quercetin exhibited dose-dependent anti-inflammatory activity in LPS-stimulated HUVECs. It effectively inhibited the production of inflammatory factors such as TNF-α, MCP-1, and IL-1β, while also down-regulating the gene and protein expression levels of ICAM-1 and VCAM-1. In conclusion, our findings indicate that there are variations in compositions among the three wolfberries, with flavonoids being the most abundant, and *in vitro* studies also confirmed the anti-inflammatory potential of quercetin. It is worth noting that *Lycium ruthenicum* contains higher levels of antioxidant components and possesses greater nutritional value, providing valuable insights for the future development and utilization of the three wolfberries.

## 1 Introduction

*Lycium*, a genus within the Solanaceae family, that mainly distributed in Gansu, Ningxia, Qinghai, Xinjiang and other areas in China (Zhang et al., 2018). At the present time, in China, seven species and three varieties of *Lycium* are recognized, which include *Lycium barbarum* L., *Lycium chinense* Mill., *Lycium ruthenicum* Murr, *Lycium dasystemum* Pojark., *Lycium truncatum* Y. C. Wang, *Lycium cylindricum* Kuang & A. M. Lu, *Lycium yunnanense* Kuang & A. M. Lu, *L. barbarum* var. *auranticarpum* K. F. Ching, *L. chinense* var. *potaninii* (Pojark.) A. M. Lu, and *L. dasystemum* var. *rubricaulium* A. M. Lu. These species and varieties are largely distributed in Gansu, Ningxia, Xinjiang, and Qinghai (Zhang et al., 2018). Wolfberry, a plant of both medicinal and culinary significance in China, and has been incorporated into the Pharmacopoeia (Chinese Pharmacopoeia Commission, 2020). Shennong’s Classic of the Thousand Materia Medica documents (Zhou and Wang, 2018): “Lord of the five internal evils, fever and thirst, weekly paralysis and wind-dampness. Consumed over time, it strengthens muscles and bones, lightens the body, delays aging, and enhances resilience to cold and heat."

The fruit of *L. barbarum* exhibits a diverse array of biological activities, encompassing antioxidation, reduction of blood sugar and blood lipid levels, anti-cancer properties, anti-aging effects, alleviation of sore waist and knees, and neuroprotection (Huang et al., 2023; Li et al., 2023; Wang et al., 2023; Yang et al., 2023; Zheng et al., 2023). Modern research has unveiled that *L. barbarum* is rich in various chemical components, such as flavonoids, alkaloids, phenolic acids, lipids, polysaccharides, amino acids, and carotenoids (Fakhfakh et al., 2022; Chen et al., 2023; Liu et al., 2023; Ma et al., 2023). In comparison to *L. barbarum*, *L. ruthenicum* boasts a higher concentration of phenols, tannins, and anthocyanins, resulting in enhanced antioxidant activity (Islam et al., 2017). *L. barbarum* var. *auranticarpum*, a variant of *L. barbarum*, while *L. barbarum* var. *auranticarpum* does not exhibit significant advantages in its active components compared to the original species, studies in 2016 and 2017 revealed its distinct characteristics, including lower levels of lycopene and polysaccharides, higher levels of flavonoids, and lower levels of carotenoids (Wang et al., 2016; Liang et al., 2017). Lu L. et al. (2019) conducted the initial evaluation of the antioxidant activity of *L. barbarum* var. *auranticarpum*, *L. barbarum*, and *L. ruthenicum*, demonstrating that *L. barbarum* var. *auranticarpum* exhibits comparable or even superior antioxidant activity to *L. barbarum*. To date, there is a scarcity of research on the metabolite compositions and interrelationships of these three wolfberries. A comprehensive understanding of the compositional makeup of these wolfberries is pivotal for their proper identification and utilization.

Research has demonstrated that wolfberry possesses notable anti-inflammatory properties, as evidenced by its extract’s capacity to mitigate the secretion and expression of TNF-α, IL-6, IL-1β, and IFN-γ, as well as to curtail the synthesis of NO (Oh et al., 2012; Song et al., 2014; Peng et al., 2019; Peng et al., 2020; Duan et al., 2024). However, the precise primary active component remains to be elucidated. Presently, the predominant constituents under investigation in wolfberry are polysaccharides and flavonoids, with quercetin being a predominant flavonoid constituent. Studies have indicated that quercetin can mitigate vascular calcification and reduce inflammatory responses (Ceccherini et al., 2024). Furthermore, quercetin has been observed to alleviate liver injury resulting from cholestasis, stress, inflammation, and fibrosis (Abushady et al., 2020). Quercetin has also been demonstrated to prevent neuronal injury by inhibiting mtROS-mediated NLRP3 inflammasome activation in microglia through the promotion of mitophagy (Han et al., 2021). Moreover, quercetin has been identified as a geroprotective agent against accelerated and natural aging in human mesenchymal stem cells (hMSCs), offering a potential therapeutic strategy for age-related disorders (Geng et al., 2019).

Metabolomics represents a burgeoning scientific domain that as a complementary field to genomics and transcriptomics, metabolomics yields invaluable insights into the physiological state of organisms. By examining the variations in metabolite profiles and metabolic pathways across diverse groups, metabolomics provides an accurate reflection of the underlying physiological transitions (Zhang et al., 2022). In a study conducted by Xie et al. (2023), the researchers conducted a comparative analysis of metabolite alterations across two distinct developmental phases of wolfberry fruits. The results indicated that although the fruits displayed similar patterns of metabolite accumulation during each developmental phase, there were discernible differences in the metabolites accumulated within the same phase. This novel insight offers a fresh perspective on the synthesis of flavonoids in wolfberries.

The research postulated that the metabolite profiles would differ across various wolfberry. Employing an extensive analytical approach with ultra-high performance liquid chromatography-tandem mass spectrometry (UPLC-MS/MS), the investigative team delineated the unique metabolites present in three distinct wolfberries. Moreover, quercetin was chosen as a potent compound to assess its anti-inflammatory properties *in vitro*. The objective of this study is to furnish essential insights into the metabolite composition of the three wolfberries, to lay a conceptual groundwork for subsequent wolfberry research, and to enhance the development of traditional Chinese medicine.

## 2 Materials and methods

### 2.1 Chemicals

Dimethyl sulfoxide (DMSO) was purchased from Sigma-Adlrich Co. (St. Louis, MO, United States). Cell Counting Kit-8 (CCK8) and Fetal Bovine Serum (FBS) were obtained from TransGen Biotech (Beijing, China). Quercetin was obtained from Solarbio science and technology Co., Ltd. (Beijing, China). Penicillin-Streptomycin and Trypsin were sourced from Gibco (Gaithersburg, MD, United States). Methanol and Acetonitrile were acquired from Merck (Darmstadt, Germany). Formic acid was obtained from Aladdin (Shanghai, China). All reagents and chemicals used in the assay were of analytical and HPLC grade.

### 2.2 Plant materials

**FIGURE 1:**
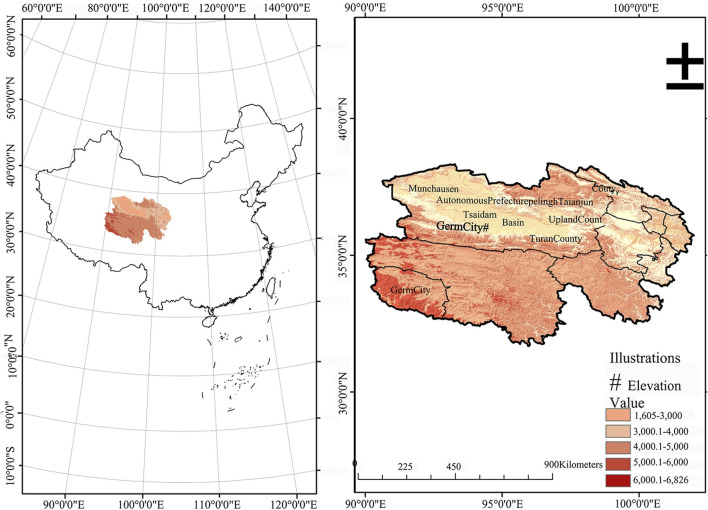
Geographical location of wolfberry.

In this study, three types of wolfberries were generously provided by Qinghai Xiangyu Agricultural Technology Co., Ltd. These wolfberries are harvested from the Nuomuhong farm, situated in Zongga Town, Dulan County, within the Haixi Tibetan and Mongolian Autonomous Prefecture of Qinghai Province. This farm is nestled in the southeastern quadrant of the Qaidam Basin, precisely at the coordinates of 96°26′4″E longitude and 36°26′46″N latitude. It is elevated at approximately 2,800 m above sea level (Figure 1). These include *L. barbarum* (RL), *L. ruthenicum* (BL), and *L. barbarum* var. *auranticarpum* (YL). RL is a cultivated wolfberry, while BL and YL are wild wolfberries.

### 2.3 Measurements of the fruit appearance index

The weight of twenty random samples of each type of wolfberry was recorded, and five samples were selected for cross-sectional photography. The transverse and longitudinal dimensions were measured, and the fruit shape index was calculated. The fruit mass (g) was determined using an electronic balance with a sensitivity of 0.001 g. The transverse and longitudinal diameters (mm) were measured using a digital Vernier Caliper with an accuracy of 0.02 mm. The fruit shape index was expressed as the ratio of the longitudinal diameter to the transverse diameter.

### 2.4 Differential metabolite profiling based on widely targeted metabolomic

#### 2.4.1 Sample extraction and preparation

The samples of wolfberries were dehydrated and dried using a vacuum freeze-drier (Scientz-100F). Subsequently, the dried samples were ground for 1.5 min at 30 Hz (MM400, Retsch). Lyophilized powder (50 mg) was then dissolved in 1.2 mL of a 70% methanol solution (1:24, w/v). The mixture was swirled every 30 min for 30 s (6 times in total) and refrigerated overnight at 4 °C. Afterward, the samples were centrifuged for 3 min at 12,000 rpm/min, and the supernatant was collected. The samples were filtered using a microporous membrane (0.22 μm pore size) and stored in the injection bottle for UPLC-MS/MS analysis.

#### 2.4.2 UPLC and ESI-QTRAP-MS/MS analysis

The analysis was conducted by MetWare (Wuhan, China). The sample extracts were analyzed using a UPLC-MS/MS system (UPLC, SHIMADZU Nexera X2; MS/MS, Applied Biosystems 4500 QTRAP). The following analytical conditions were employed: UPLC: column-Agilent SB-C18 (1.8 µm, 2.1 mm*100 mm); phase A-ultra-pure water containing 0.1% formic acid; phase B-acetonitrile containing 0.1% formic acid. The sample measurements were performed with a gradient program, starting with 95%A and 5%B. Within 9 min, the proportion of phase B increased to 95% and remained constant for 1 min. The proportion of phase A was reduced to 5% in the 10.00–11.10 min range and equilibrated at 5% until 14 min. The flow rate was set at 0.35 mL/min, the column temperature was maintained at 40°C, and the injection volume was 4 μL. The effluent was then connected to an ESI-triple quadrupole linear ion trap (QTRAP)-MS (Heinrich et al., 2022).

The QTRAP-MS, AB4500 QTRAP UPLC/MS/MS system with ESI Turbo Ion-Spray interface, was used to perform scans in both positive and negative ion mode. The scans were conducted using the linear ion trap (LIT) and triple quadrupole (QQQ) modes, and the data was analyzed using Analyst 1.6.3 software. The ESI source operation parameters were set as follows: ion source temperature at 550 °C, ion spray voltage (IS) at 5500 V in positive ion mode and −4500 V in negative ion mode. The ion source gas I (GSI), gas II (GSII), and curtain gas were set at 50, 60, and 25 psi respectively, and the collision activated dissociation (CAD) was set to high. For instrument tuning and mass calibration, 10 and 100 μM/L polypropylene glycol solutions were used in QQQ and LIT modes respectively. The QQQ scans used MRM experiments with nitrogen as the collision gas set to medium. Further optimization of declustering voltage (DP) and collision energy (CE) was performed for each MRM ion pair. A specific set of MRM ion pairs were monitored for each period based on the eluted metabolites (Heinrich et al., 2022).

#### 2.4.3 Multivariate statistical analyses

In the multivariate statistical analyses, hierarchical cluster analysis (HCA), principal component analysis (PCA), and orthogonal partial least squares discriminant analysis (OPLS-DA) were conducted on the metabolic data of each sample. PCA was used to display the original state of the metabolomic data and to provide variable information. The built-in statistical prcomp function of R software (https://www.r-project.org/) was used to analyze the data. The OPLS-DA model was performed using the R software package Metob Analyst R to compare the metabolic characteristics of different wolfberry. The metabolite data were log_2_-transformed, and then OPLS-DA analysis was conducted. Differential metabolites were screened based on a variable importance projection (VIP) ≥ 1 in the OPLS-DA model and a differential multiple value (Fold change) ≥ 2 or ≤0.5. A Venn diagram was used to represent the number of different metabolites.

#### 2.4.4 KEGG annotation and enrichment analysis

The metabolites were annotated using the KEGG compound database (https://www.kegg.jp/kegg/compound/) and mapped to the KEGG pathway database (https://www.kegg.jp/kegg/pathway.html). Metabolite enrichment analysis was conducted on pathways with significantly regulated metabolites, and their significance was assessed using the *p*-values obtained from hypergeometric tests.

### 2.5 *In Vitro* anti-inflammatory experiments

#### 2.5.1 Cell culture

The HUVECs were acquired from the esteemed biotechnology development firm, Qingflag, located in Shanghai, China. HUVECs were cultured in RPMI1640 medium (Biosharp, Anhui, China) supplemented with 10% fetal bovine serum (FBS) (TransGen Biotech, Beijing, China) and 1% penicillin/streptomycin (P/S, Gibco, United States) in a 37 °C incubator with 5% CO_2_.

#### 2.5.2 Cell viability analysis

HUVECs were seeded in 96-well plates (5×10^5^/mL), cultured overnight, and treated with different concentrations of quercetin for 24 and 48 h when the cells reached 60%–70% confluency, where NC is the control group. The original culture medium was discarded, and the cells were washed twice with PBS. The basal medium and CCK-8 mother solution were mixed in a ratio of 10:1 and 100 μL of the mixture was added to each well. The plate was then incubated at 37 °C in the dark for 30 min. The absorbance at 450 nm was measured using a microplate reader.

#### 2.5.3 Anti-inflammatory effects

The anti-inflammatory effect of quercetin was assessed *in vitro* by measuring the cellular inflammatory factor. HUVEC cells were pre-inoculated in a 6-well plate (5×10^5^/mL) and cultured overnight. After being treated with LPS for 6 h and different concentrations of quercetin for 48 h, the supernatant was collected and placed on ice. Following the operation instructions, the kit was allowed to equilibrate at room temperature for 15–30 min. The test solution and standard sample were added to the enzyme-coated plate, followed by incubation with the enzyme solution at 37°C for 60 min. The plate was then washed and the chromogenic solution was added, followed by incubation at 37°C away from light for 15 min. Finally, the terminating solution was added for determination. Anti-inflammatory factors were purchased from BYabscience, Nanjing, China (number: 33,130, 29,076, 33,118, 28,113).

#### 2.5.4 Quantitative real-time PCR analysis

Total RNA was extracted from the cultured cells using the TransGen Biotech RNA extraction kit, and the RNA concentration and integrity were assessed. The PrimeScript®RT Mix was used to reverse 1 μg of total RNA into cDNA for mRNA detection, and PerfectStart Green qPCR Mix was used for qPCR analysis. The gene expression level was calculated using the 2^−ΔΔCt^ method and normalized to GAPDH mRNA expression level. The primers used were as follows:

GAPDH-forward: 5′-AGA​TCC​CTC​CAA​AAT​CAA​GTG​G-3′

GAPDH-reverse: 5′-GGC​AGA​GAT​GAT​GAC​CCT​TTT-3′

VCAM-1- forward: 5′-GAT​TCT​GTG​CCC​ACA​GTA​AGG​C-3′

VCAM-1- reverse: 5′-TGG​TCA​CAG​AGC​CAC​CTT​CTT​G-3′

ICAM-1- forward: 5′-AGC​GGC​TGA​CGT​GTG​CAG​TAA​T-3′

ICAM-1- reverse: 5′-TCT​GAG​ACC​TCT​GGC​TTC​GTC​A-3′

#### 2.5.5 Western blot

Protein was extracted from the cells using protein extraction reagents according to the relevant instructions, and the total protein concentration was measured using the BCA protein assay kit (Beijing Solarbio Science & Technology Co., Ltd.). The prepared loaded protein was subjected to SDS-PAGE, then transferred to a PVDF membrane, which was subsequently incubated overnight at 4°C with appropriate primary antibodies (Cell Signaling Technology, VCAM-1: 13,662, ICAM-1: 4915, GAPDH: 5174). Conjugation was performed using horseradish peroxidase-conjugated secondary antibodies (Beijing Solarbio Science & Technology Co., Ltd., HS101-01). Color development was achieved using ECL luminescent liquid.

### 2.6 Statistical analysis

All analysis results were expressed as the mean ± standard deviation (SD) of three replicates. Using IBM SPSS 20 for multi-group comparisons, using one-way ANOVA, *p* < 0.05 was considered different. GraphPad Prism 8.0.2 software was used for data visualization.

## 3 Results

### 3.1 Appearance index of three different accessions of wolfberry

**FIGURE 2:**
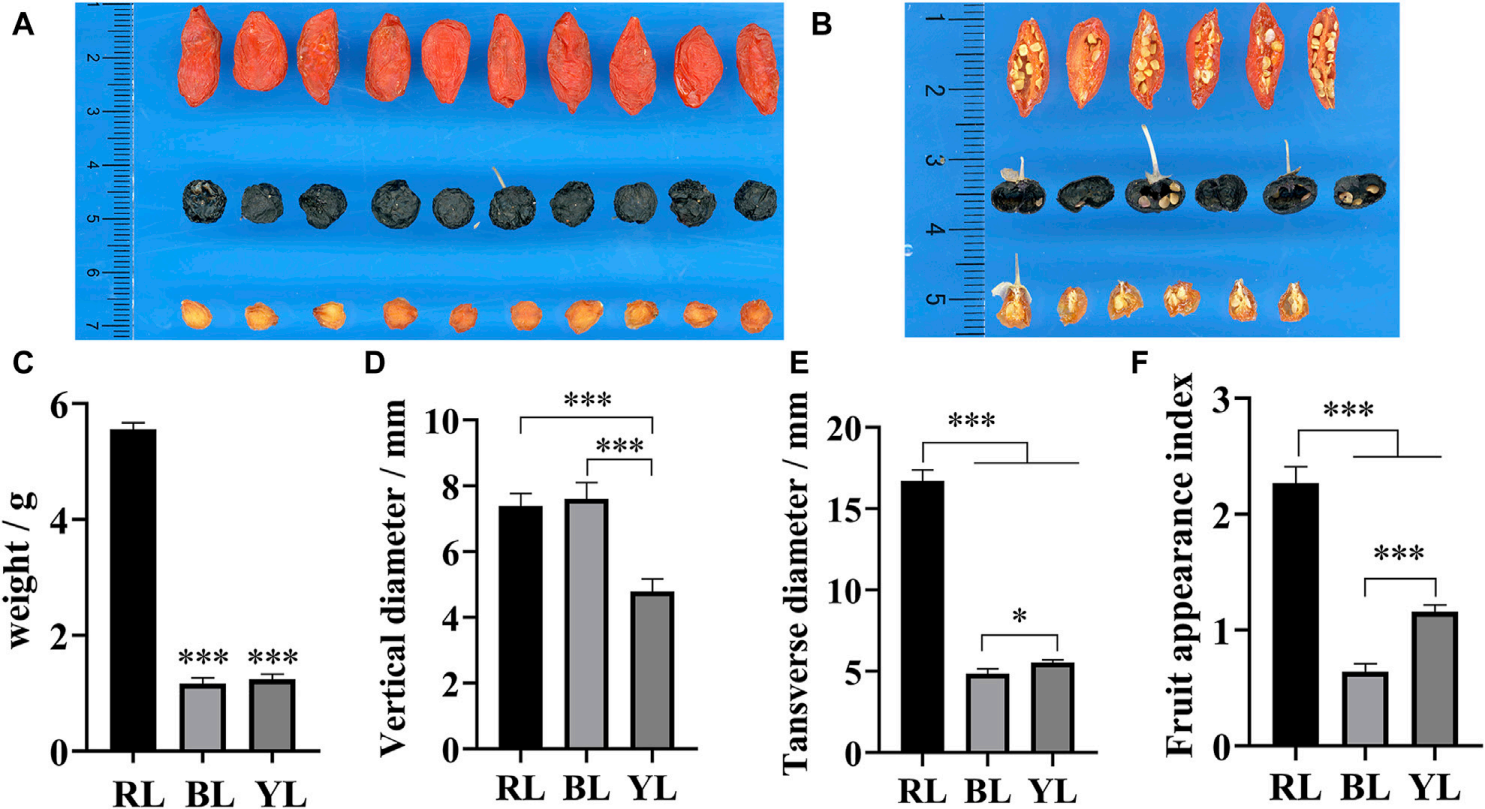
Appearance and cross-section of different wolfberries fruits. **(A)** Appearance of wolfberries fruit; **(B)** cross-section of wolfberries fruit; **(C)** weight of wolfberries fruit; **(D)** transverse diameter of wolfberries fruit; **(E)** longitudinal diameter of wolfberries fruit; **(F)** the ratio of longitudinal to transverse diameter of wolfberries fruit. * and *** represent a significant correlation at the *p* < 0.05 and *p* < 0.001 levels, respectively.

Among the three different wolfberry accessions, RL exhibited the largest physical characteristics, including appearance, single fruit weight, longitudinal diameter, and transverse diameter (Figures 2A–D). Furthermore, the weight of RL was significantly higher than that of BL and YL. In terms of appearance morphology, BL was larger than YL, but its weight was smaller in comparison. A cross-sectional view revealed that BL appeared hollow, whereas the pulp was still present in the center of YL (Figure 2B). This disparity is the primary reason for YL being heavier than BL. The appearance index demonstrated the following order: RL>YL>BL (Figure 2F). These findings suggest that the fruit composition of YL and RL may be more similar, while BL differs from them.

### 3.2 Characterization of metabolite components in three wolfberries through widely targeted metabolomics

#### 3.2.1 Qualitative and quantitative analysis of metabolites in three wolfberries

**FIGURE 3:**
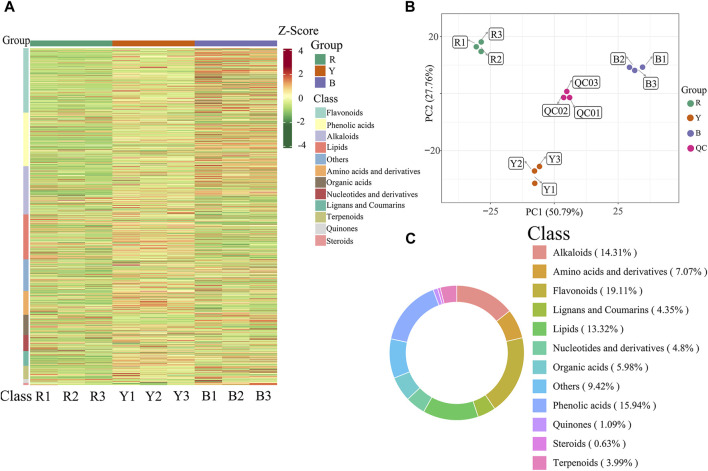
Heatmap of the metabolites under different wolfberries **(A)**, the PCA score plot **(B)**, the composition of the metabolites of wolfberry fruits **(C)**. In the PCA score plot, the abscissa is the first principal component PC1, the ordinate is the second principal component PC2. Each point represents a sample, and different groups are marked with different colors. The number in parentheses is the score of the principal component, which indicates the percentage of the overall variance explained by the corresponding principal component.

**FIGURE 4:**
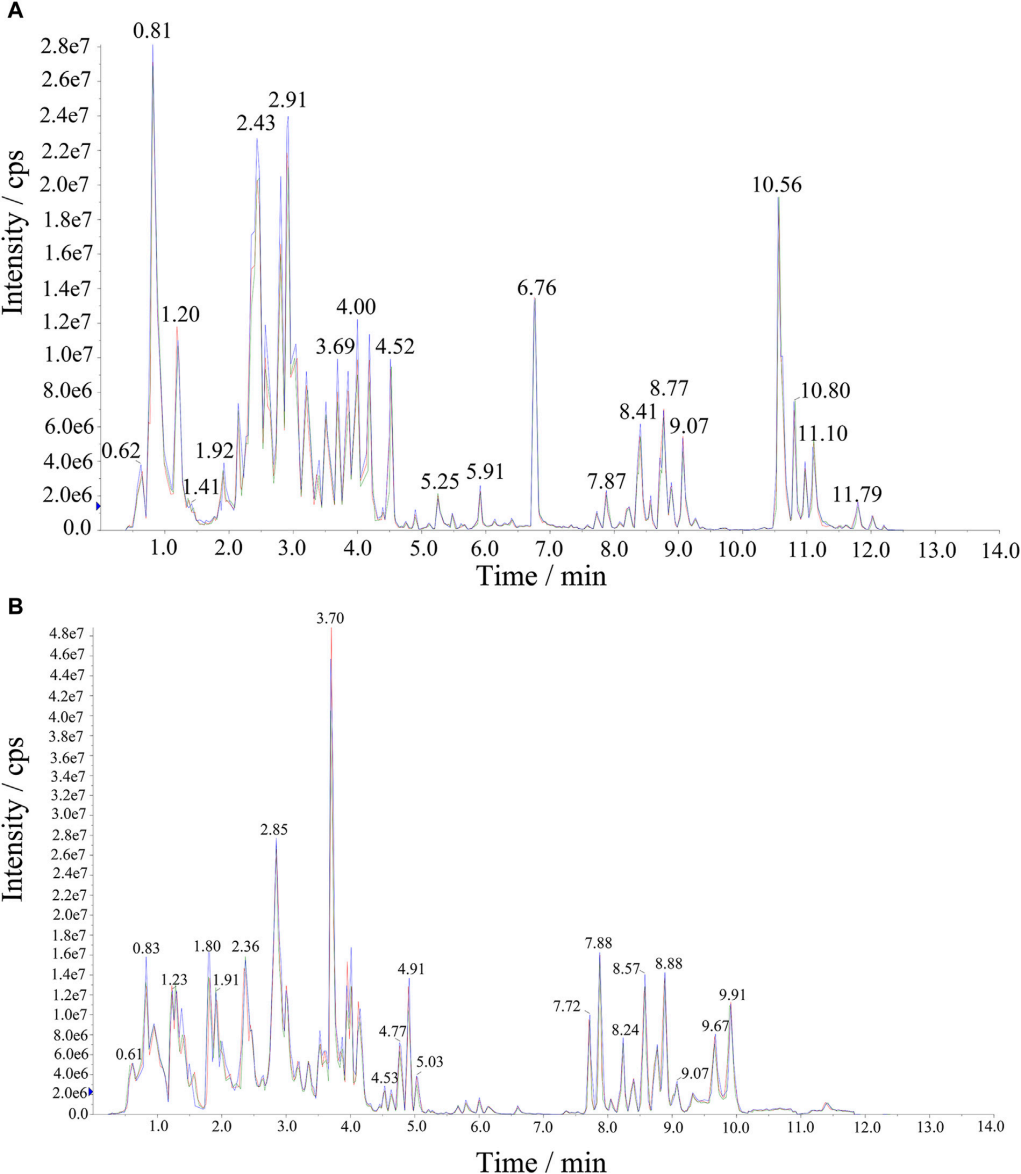
The extracted ion chromatograph (XIC) of QC sample. **(A)** Negative Ion Mode, **(B)** Positive Ion Mode.

Significant variations in the metabolite content among the three dried wolfberry samples were observed through a comprehensive and targeted metabolomic analysis (Figure 3A). A total of 1,104 metabolites were identified using characteristic screening, with 559 metabolites in positive ion mode and 545 in negative ion mode. These metabolites were classified into 12 main groups, including amino acids and derivatives, phenolic acids, nucleotides and derivatives, flavonoids, lignans and coumarins, alkaloids, terpenoids, organic acids, steroids, lipids, quinones, and others. Among these categories, flavonoids were the most abundant, comprising 211 metabolites, which accounted for 19.11% of the total metabolites (Figure 3C). Flavonoid compounds, such as Chalcone, dihydro-flavonoids, dihydroflavonols, flavonols, flavanols, anthocyanins, and others, exhibited varying content trends in the three types of wolfberries: BL>YL>RL. These findings highlight significant differences in the metabolic profiles among the three sample sets. The total ion flow maps (TIC plots) of the quality control (QC) samples displayed a high level of overlap, indicating the reliability and repeatability of the results (Figure 4).

#### 3.2.2 Multivariate statistical analysis

A multivariate statistical analysis was conducted on three different types of wolfberries, revealing variations in metabolites. Unsupervised principal component analysis (PCA) was employed to assess the overall dissimilarities among the samples (Figure 3B), with highly concentrated features indicating better repeatability within each group. PCA provides insights into the overall metabolic differences between the groups and the extent of variation among samples within each group. The results demonstrated that PC1 and PC2 accounted for 50.79% and 27.76% of the variance, respectively. PC1 and PC2 effectively distinguished the three types of wolfberries, suggesting variations in metabolite accumulation among different varieties.

The PCA analysis results revealed that the metabolites of the three types of wolfberries could be classified into three distinct groups, indicating significant differences in the metabolite profiles of each group.

#### 3.2.3 Identification of differential metabolites

**FIGURE 5:**
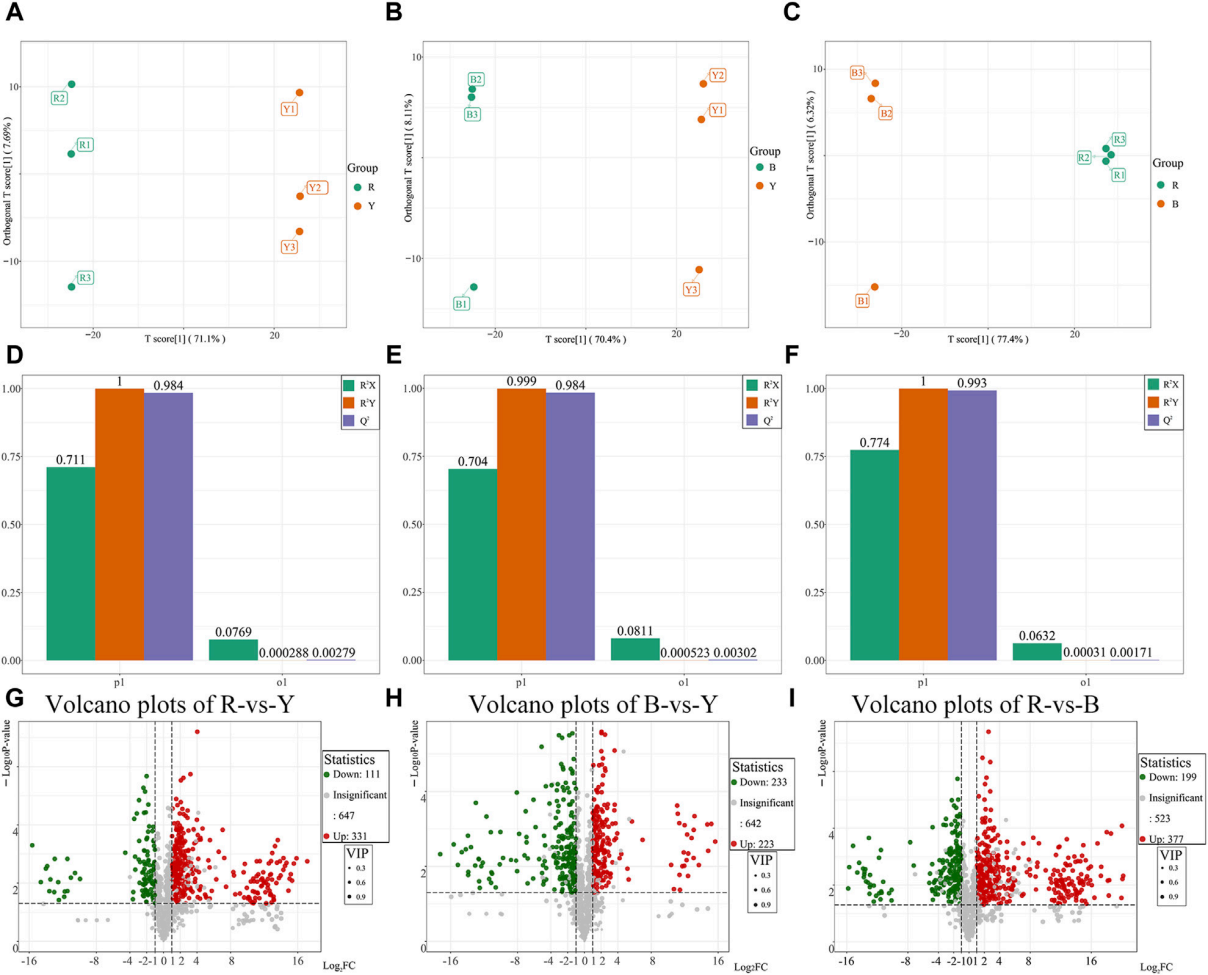
Differential metabolites analysis and Venn diagram of different wolfberries. **(A–C)**: Score plots generated from OPLS-DA in RL vs. YL groups **(A)**, the BL vs. YL groups **(B)**, the RL vs. BL group **(C)**. **(D–F)**: OPLS-DA verification diagram between RL vs. YL groups **(D)**, the BL vs. YL groups **(E)**, the RL vs. BL group **(F)**. **(G–I)**: Volcano plots showing the differential metabolites expression levels between RL vs. YL groups **(G)**, the BL vs. YL groups **(H)**, the RL vs. BL group **(I)**.

Orthogonal partial least squares discriminant analysis (OPLS-DA) was utilized to identify distinct metabolites and effectively demonstrate the differences between the three wolfberries by filtering out orthogonal variables in metabolites that are unrelated to categorical variables. The OPLS-DA model was employed to compare the pairwise differences among the three wolfberries and identify the metabolites responsible for these differences. The OPLS-DA model successfully dispersed the scores of the groups (Figures 5A–C). To validate the OPLS-DA model, 200 alignment experiments were conducted, and the results indicated that both R^2^Y and Q^2^ scores exceeded 0.9, confirming the suitability of the model (Figures 5D–F). This robustness and reliability of the OPLS-DA model were demonstrated, leaving no room for doubt.

Based on the results of the OPLS-DA model, different metabolites were selected between the various groups. The differential metabolites in the three wolfberries were screened using a threshold of VIP ≥1, FC ≥ 2 or FC ≤ 0.5 and *p* < 0.05. The results were then visualized using volcano maps and Venn plots (Figures 5G–I). In comparison to RL, YL exhibited 331 upregulated and 111 downregulated metabolites, including 59 upregulated and 13 downregulated flavonoids, respectively. When comparing YL and BL, 223 metabolites showed upregulation, including 33 flavonoids, while 233 metabolites showed downregulation, including 78 flavonoids. Between RL and BL, there were 377 differential expressions, including 87 flavonoids.

**FIGURE 6:**
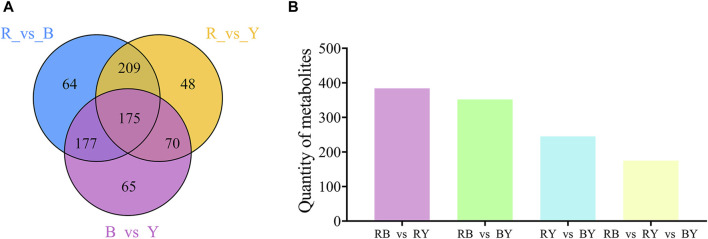
Venn diagram **(A)** and histogram **(B)** illustrating the overlapping and specific differential metabolites for three comparison groups (RL vs. BL, RL vs. YL, BL vs. YL).

The Wayne diagram was used to illustrate the relationship between different metabolites in the BL vs. RL, BL vs. YL, and YL vs. RL comparisons. In these three paired comparisons, a total of 175 identical differential metabolites were identified (Figure 6). These metabolites included amino acids and their derivatives (3), phenolic acids (41), nucleotides and their derivatives (1), flavonoids (45), quinones (2), lignans and coumarins (13), alkaloids (35), terpenes (10), organic acids (5), lipids (12), and others (8). Among the various compounds found in wolfberries, a significant concentration is present in black wolfberries, which include betulinic acid, rhodioloside, chlorogenic acid C, charanthin, isoquercetin, rutin, genistein, indole, and orcinol. A lesser quantity of these compounds is found in yellow wolfberries, such as linoleic acid, taraxacic acid, piperine, dauricine, L-theanine, D-sorbitol, and 5-hydroxytryptamine. Red wolfberries, on the other hand, contain higher levels of cinnamic acid, khellin, syringaresinol B, and alpha-bisabolol.

### 3.3 Functional annotation and enrichment analysis of differential metabolites and analysis of KEGG metabolic pathway

To further analyze the functional annotation and enrichment of the differential metabolites, as well as the KEGG metabolic pathway analysis, we utilized the KEGG database, which is a widely used public pathway database for studying signal transduction pathways and metabolite accumulation (Kanehisa and Goto, 2000). KEGG, which stands for Kyoto Encyclopedia of Genes and Genomes, provides valuable information for this type of analysis (Nucleic Acids Research, 27–30).

**FIGURE 7:**
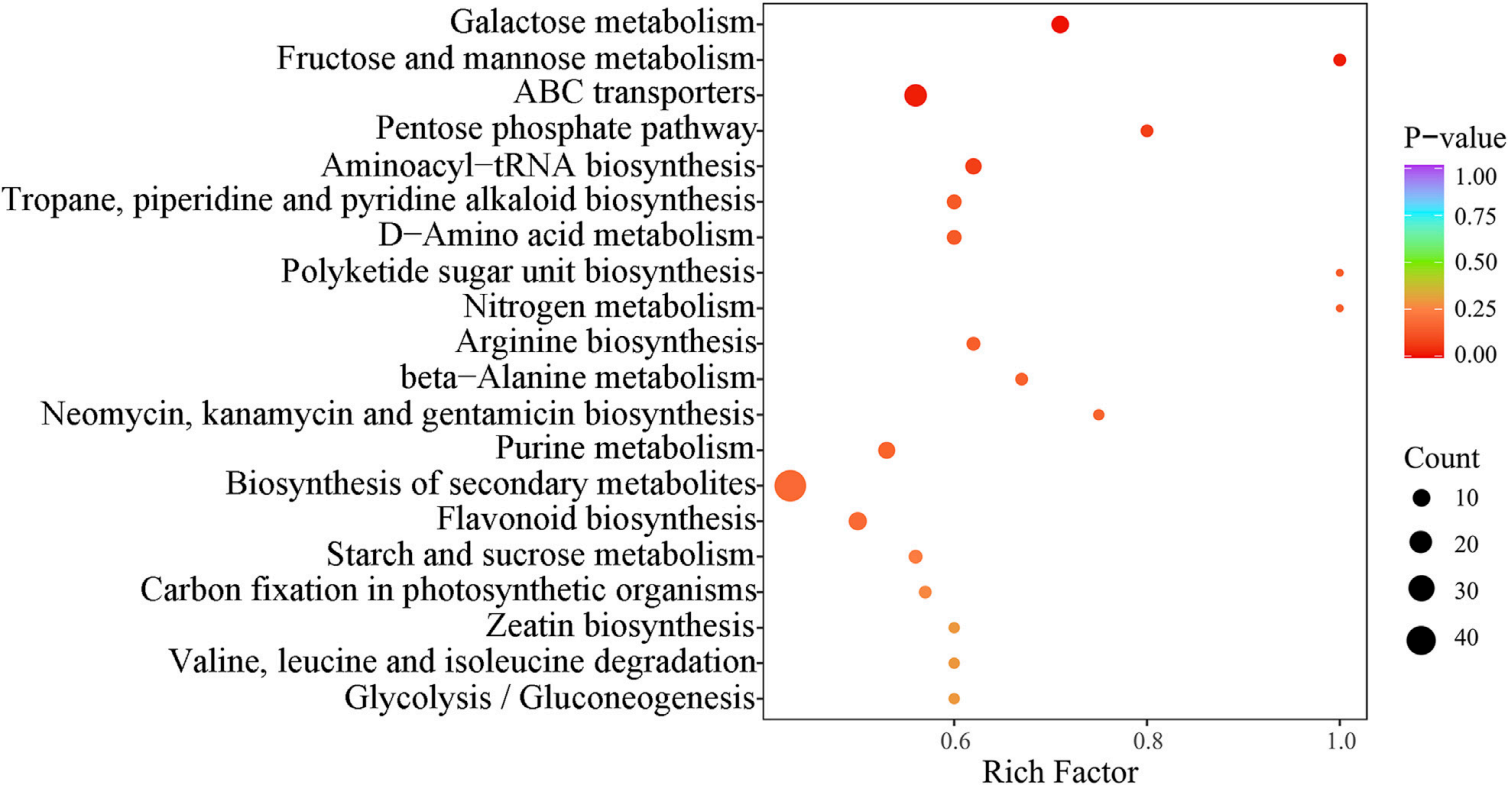
KEGG pathway analysis of differential metabolites for three wolfberries. In the plot, each bubble (which represents a metabolic pathway) and abscissa indicate the size of the factors affecting the pathway (bigger bubbles represent bigger impacts). Bubble color indicates the *p*-value of the enrichment analysis. Lighter colors indicate lower enrichment.

**FIGURE 8:**
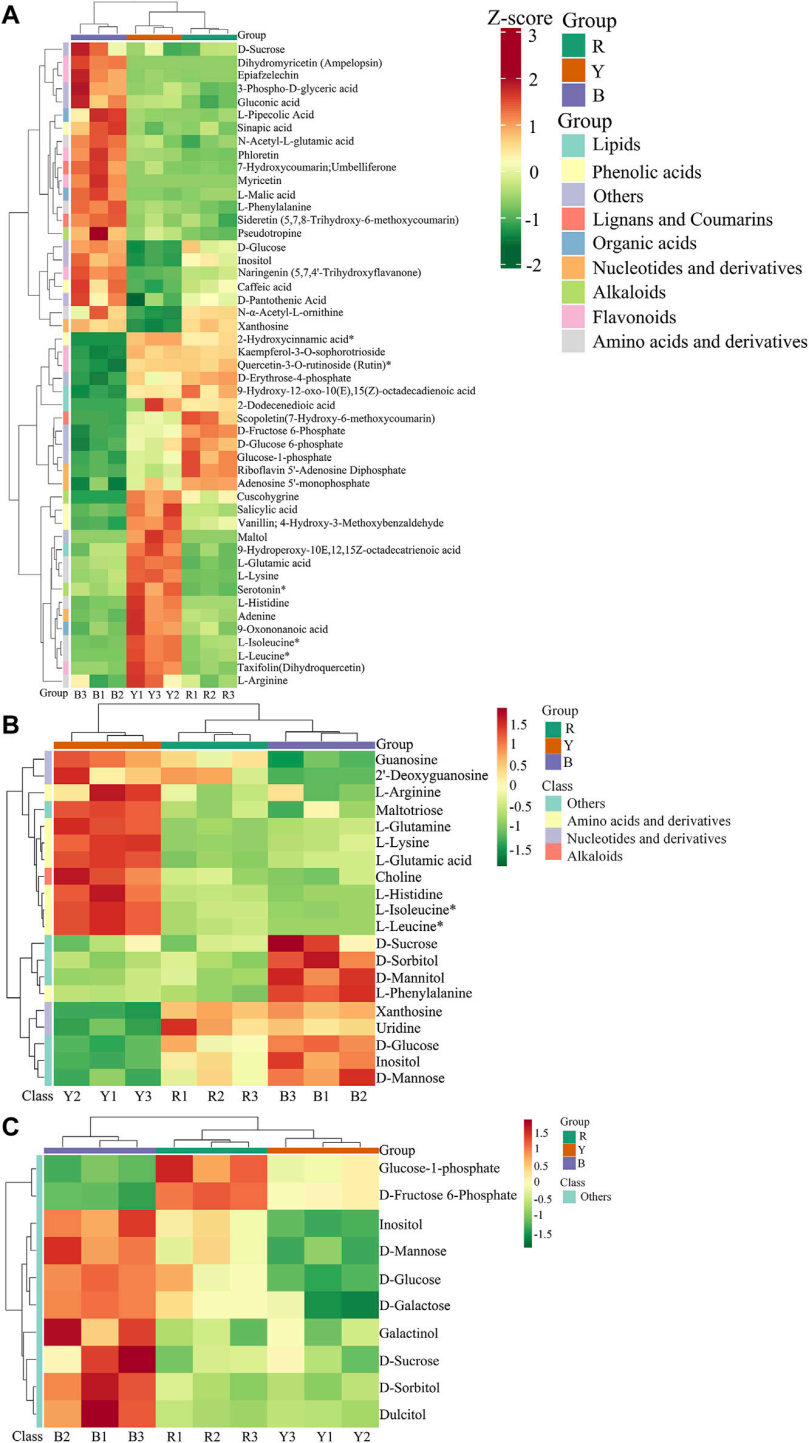
Three metabolic pathways. **(A)** Biosynthesis of secondary metabolites. **(B)** ABC transport. **(C)** Galactose metabolism.

In this study, we performed KEGG enrichment analysis on the detected compounds. By integrating the analysis of the differential metabolites identified in both positive and negative ion modes, we were able to annotate and display the functions of these metabolites. A total of 70 metabolic pathways were integrated, covering 431 metabolites. The top 20 pathways were identified, with a particular focus on secondary metabolite biosynthesis, ABC transport, and galactose metabolism. These pathways enriched 49, 20, and 10 metabolites, respectively (Figures 7, 8).

The analysis of the aforementioned data has revealed that three wolfberries contain the highest concentration of flavonoids, with quercetin and its glycosylated derivatives being the most prevalent. Nonetheless, due to the intricate nature of their separation and the limited availability of standard products, quercetin a commonly recognized flavonoid was chosen for the anti-inflammatory assessment. It is postulated that the therapeutic properties of wolfberries may be attributed, in part, to the presence of the flavonoid quercetin.

### 3.4 *In vitro* anti-inflammatory effect of quercetin

#### 3.4.1 Assessment of cell cytotoxicity

**FIGURE 9:**
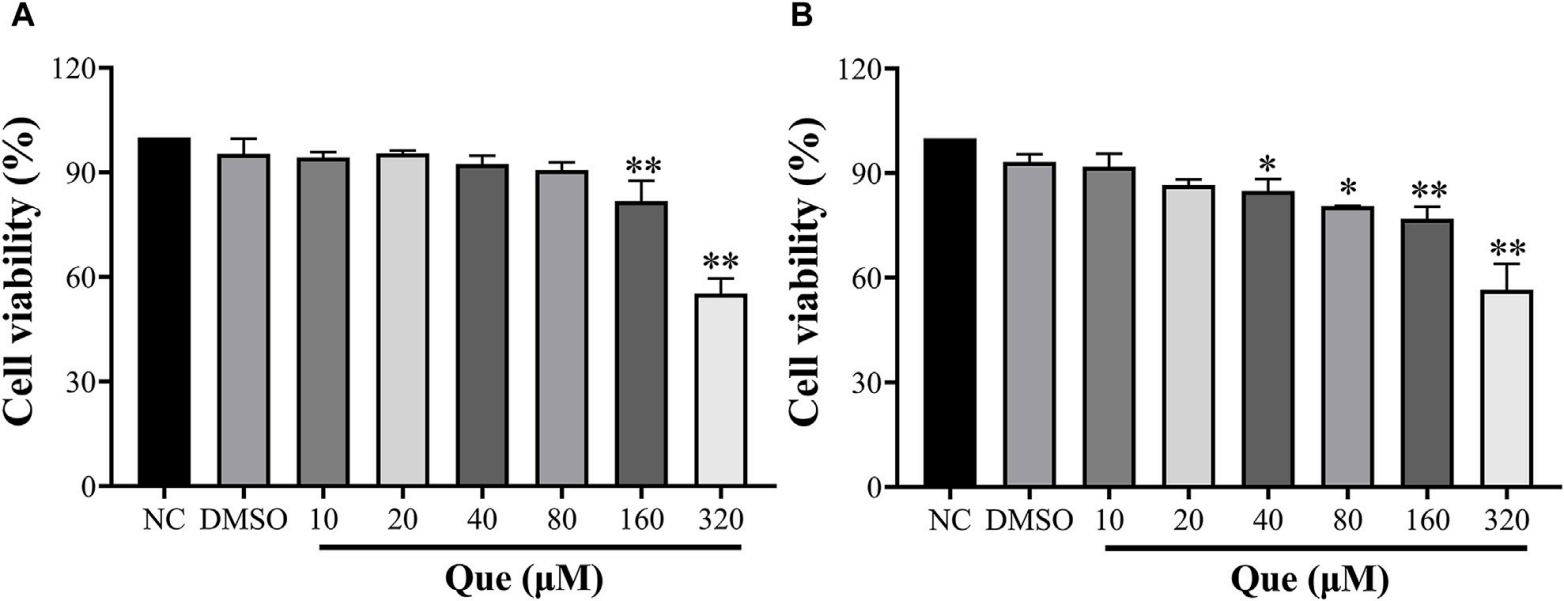
Effect of Quercetin on HUVEC viability. Cell viability was determined using the CCK-8 assay, and optical densities at 450 nm are shown. **(A)**: 24 h, **(B)**: 48 h. Data are expressed as the means ± SDs. *p < 0.05 compared to the untreated group.

In order to investigate the potential cytotoxicity of quercetin, HUVECs were exposed to different concentrations of quercetin (10, 20, 40, 80, 160, 320 μM) for 24 and 48 h to assess cell viability. The findings of this study indicated that quercetin exhibited no toxic effects on HUVECs even at a high concentration of 80 μM after 24 h, with cell viability remaining above 90%. This suggests that quercetin does not hinder the normal functioning of the cells. However, at a concentration of 160 μM, there was a significant decrease in cell viability compared to the untreated group. Additionally, after 48 h, a concentration of 40 μM demonstrated an inhibitory effect on HUVECs (Figure 9).

#### 3.4.2 Quercetin inhibited LPS-induced pro-inflammatory cytokine production in HUVECs

**FIGURE 10:**
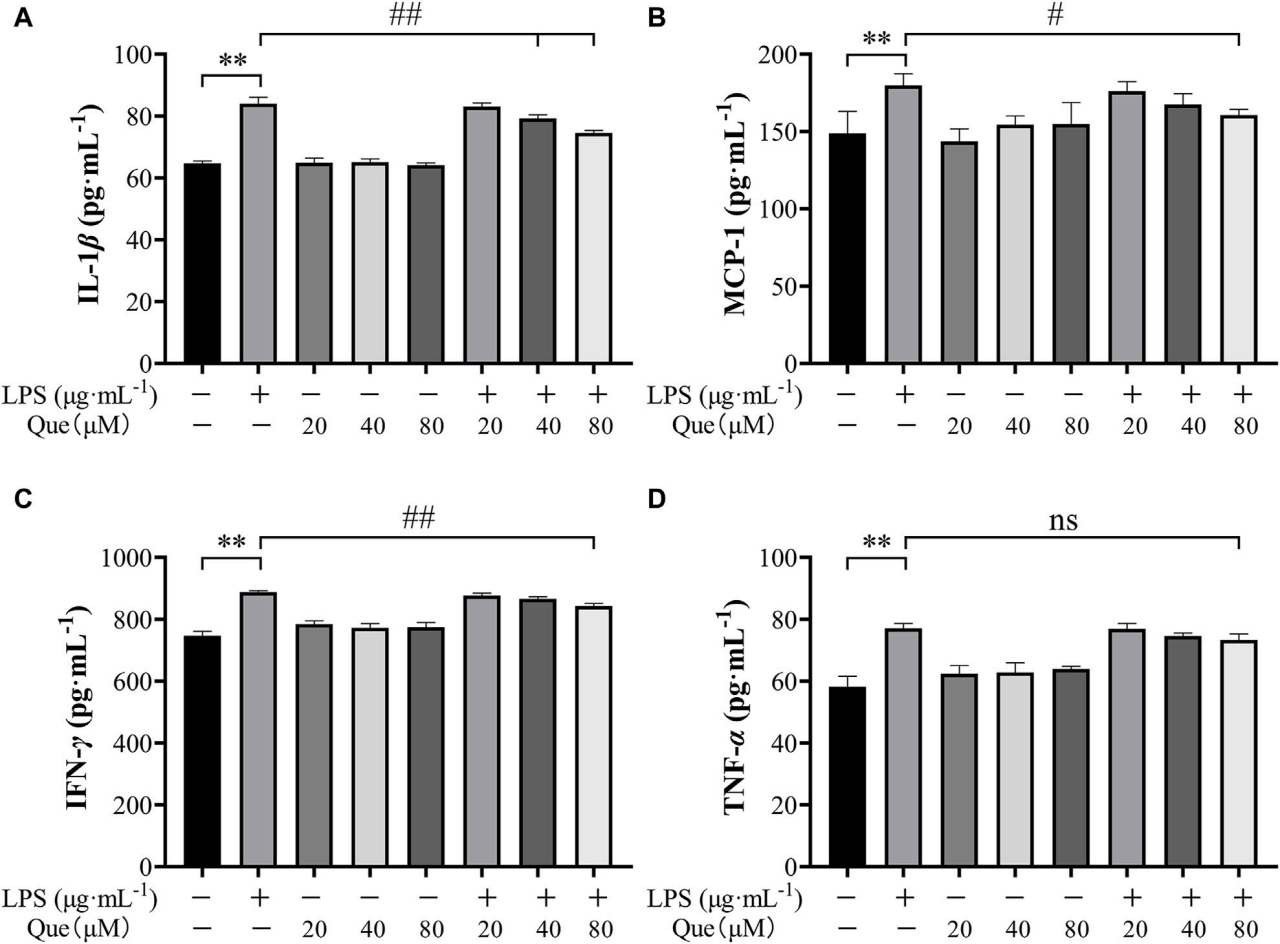
The pharmacological action of quercetin on TNF-α, IL-1β, IFN-*γ* and MCP-1 levels in LPS- induced HUVEC cells. **(A)** Amount of IL-1β in the supernatant of cells pretreated with quercetin. **(B)** Amount of MCP-1 in the supernatant of cells pretreated with quercetin. **(C)** Amount of IFN-*γ* in the supernatant of cells pretreated with quercetin. **(D)** Amount of TNF-α in the supernatant of cells pretreated with quercetin. Results were expressed as the average value ± SD. ##*p* < 0.01 indicates that it is different from the model group. *p < 0.05 and **p < 0.01, indicating that there is a significant difference with the control group.

Quercetin was found to inhibit the production of pro-inflammatory cytokines in HUVECs induced by LPS. The experimental results revealed a significant increase in the levels of TNF-α, IL-1β, MCP-1, and IFN-*γ* in the supernatant of LPS-induced HUVECs. However, pretreatment with quercetin resulted in a reduction in the secretion of these inflammatory factors (Figures 10A–D). As depicted in Figures 10A–D, the levels of inflammatory factors in the supernatant of LPS-induced HUVECs were significantly different from those in the control group (*p* < 0.01). Notably, at quercetin concentrations of 40 μM and 80 μM, there was a significant decrease in the amount of IL-1β (Figure 10A), demonstrating a significant difference compared to the model group (*p* < 0.01). At a quercetin concentration of 80 μM, the secretion of MCP-1 and IFN-*γ* in the cell supernatant also decreased (Figures 10B, C), showing a significant difference compared to the model group (*p* < 0.05). Although the concentrations of quercetin (20 μM, 40 μM, and 80 μM) did not lead to a significant difference in TNF-α levels compared to the model group, there was a tendency for a decrease.

#### 3.4.3 Effect of quercetin on the gene expression of *ICAM-1* and *VCAM-1* in HUVECs induced by LPS

**FIGURE 11:**
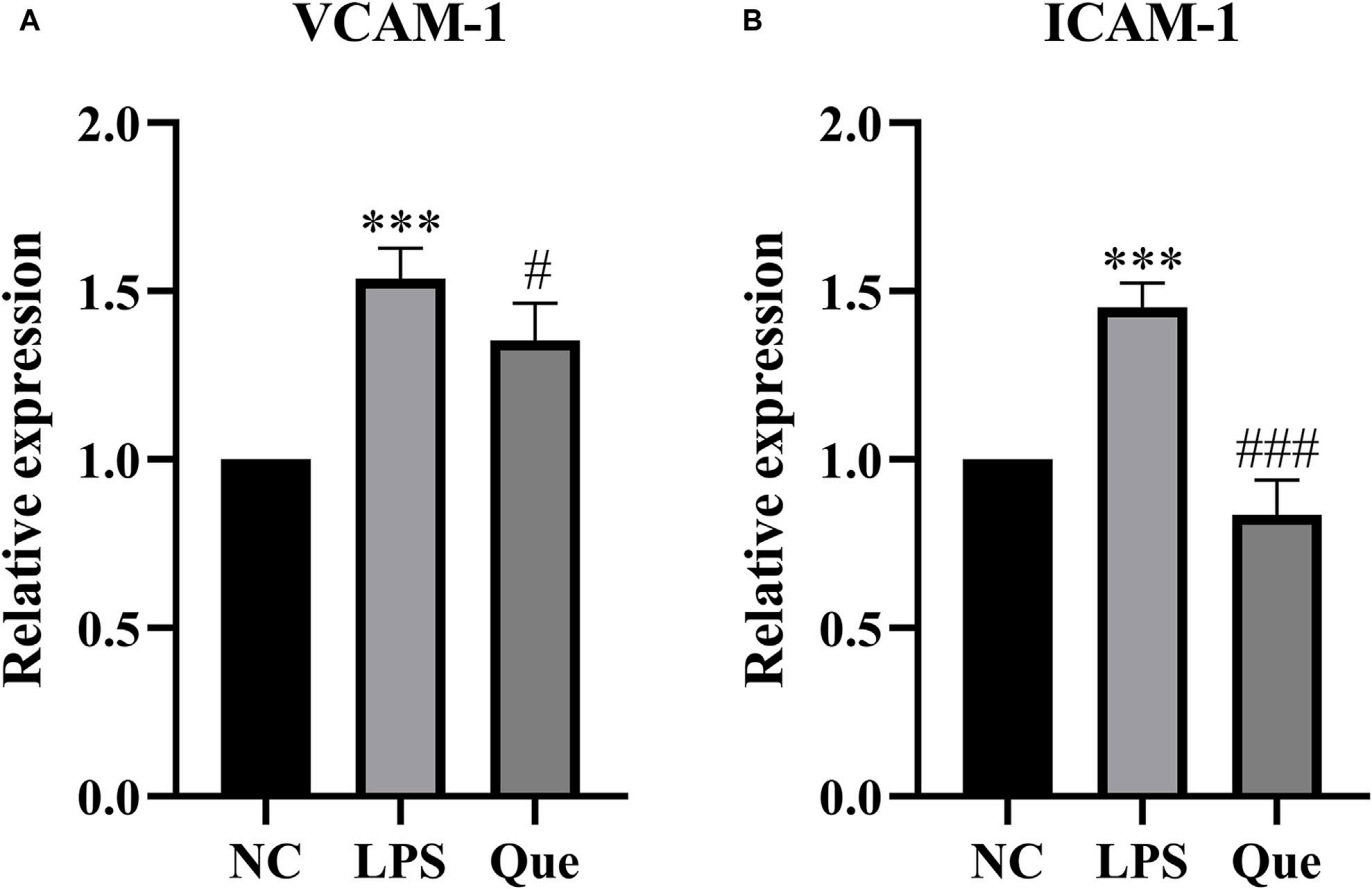
The effect of LPS exposure on the expression of VCAM-1 **(A)** and ICAM-1 **(B)** genes in HUVEC cells. #p indicates that it is different from the model group. *p indicates that there is a significant.

The effect of quercetin on the gene expression of *ICAM-1* and *VCAM-1* in HUVECs induced by LPS was investigated. *ICAM-1* and *VCAM-1* are crucial signaling molecules in endothelial cells that function as cell adhesion factors and can be activated by inflammatory mediators. Figure 11 demonstrates that compared to the normal group, the expression of *ICAM-1* and *VCAM-1* mRNA increased in the LPS group. However, upon the addition of quercetin of 40 μM, the expression of *ICAM-1* and *VCAM-1* mRNA decreased. These findings suggest that quercetin has the ability to inhibit LPS-induced cellular inflammation.

#### 3.4.4 Effect of quercetin on ICAM-1 and VCAM-1 protein expression in HUVECs induced by LPS

**FIGURE 12:**
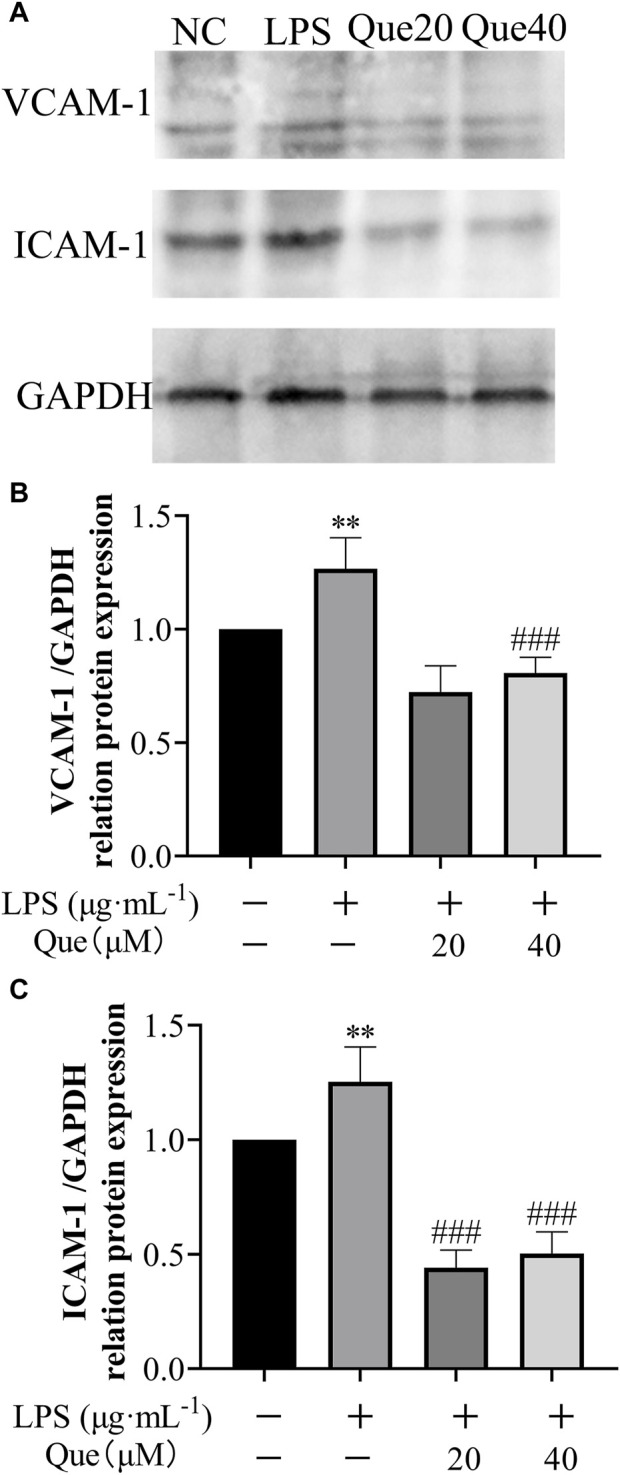
The effect of LPS exposure on the expression of VCAM-1 and ICAM-1 proteins in HUVEC cells. **(A)** protein blotting results of VCAM-1, ICAM-1 and GAPDH; **(B)** relative grey values of VCAM-1 calculated using GAPDH as control; **(C)** relative grey values of ICAM-1 calculated using GAPDH as control. #p indicates that it is different from the model group. *p indicates that there is a significant difference with the control group.

Furthermore, the effect of quercetin on the protein expression of ICAM-1 and VCAM-1 in HUVECs induced by LPS was examined (Figure 12). Following treatment with quercetin, the expressions of VCAM-1 and ICAM-1 proteins in the 20 μM (Que20) and 40 μM (Que40) groups showed significant differences compared to the model group (*p* < 0.001). This suggests that quercetin possesses the capability to suppress inflammation caused by LPS.

## 4 Discussion

In contemporary society, amidst the growing quest for a healthy lifestyle and a rekindled appreciation for the benefits of traditional Chinese medicinal herbs, wolfberry has garnered significant respect for its rich nutritional value and pharmacological properties. Research has illuminated that the nutritional profile and pharmacological effects of wolfberry are subject to a multitude of influencing factors, such as cultivation practices and species variations. Within China, scholarly attention has been particularly focused on the genus *Lycium*, with emphasis on *L. ruthenicum* and *L. barbarum*. Empirical evidence has substantiated that *L. ruthenicum*, *L. barbarum*, and *L. barbarum* var. *auranticarpum* exhibit distinct nutritional compositions, with *L. ruthenicum* emerging as the most nutrient-dense (Zhou et al., 2021). Moreover, the antioxidant prowess of *L. ruthenicum* surpasses that of *L. barbarum*, conferring greater health advantages to humans (Song et al., 2021). This study has revealed that *L. ruthenicum* harbors a more abundant array of flavonoids and phenolic acids, as well as the highest concentration of antioxidant constituents, indicating that *L. ruthenicum* may possess superior antioxidant capabilities. This aligns with the findings of Du et al. (2024), who noted the heightened antioxidant activity and flavonoid content in *L. ruthenicum* sourced from Qinghai. Furthermore, the lower lipid content of *L. ruthenicum* may render it a more suitable choice for consumption by individuals adhering to high-fat diets.

As a relatively unexplored germplasm resource, *L. barbarum* var. *auranticarpum* has garnered minimal scholarly attention and research into its bioactive compounds, largely due to its diminutive fruit size, reduced yield, and susceptibility to damage upon desiccation. Our study revealed that *L. barbarum* var. *auranticarpum* not only boasts a higher lipid content but also contains elevated levels of phenolic acids, flavonoids, amino acids, and organic acids, particularly essential amino acids such as glutamic acid and lysine. These findings suggest that *L. barbarum* var. *auranticarpum* may possess a superior nutritional profile compared to its counterpart *L. barbarum*. This conclusion diverges from previous studies (Liang et al., 2019), potentially attributed to the utilization of wild-harvested *L. barbarum* var. *auranticarpum* in our experiments. Furthermore, our analysis indicated that *L. barbarum* var. *auranticarpum* exhibits higher concentrations of adenosine 5-phosphate and guanosine 5-phosphate, compounds known to contribute to the distinctive flavor of wolfberry fruits (Gong et al., 2016; Manninen et al., 2018). Notably, the fresh fruit juice yield of *L. barbarum* var. *auranticarpum* was observed to be significantly greater than that of *L. barbarum* (Wang et al., 2016), which could explain the superior taste of its fresh produce. Despite these attributes, the predominant market for wolfberry fruits in China favors dried fruit forms, and the fragile skin of *L. barbarum* var. *auranticarpum* leads to unsatisfactory appearance post-drying, negatively impacting sales. Consequently, the development of *L. barbarum* var. *auranticarpum* remains in its nascent stage. Nevertheless, given its high nutritional value, the processing of fresh wolfberry juice and the cultivation of improved fresh food varieties represent the emerging trends and focal points in the research of *L. barbarum* var. *auranticarpum*.

Research indicates that wolfberry extracts possess a range of pharmacological properties. Specifically, *L. barbarum* fruit extract has been observed to lower levels of TNF-α and IL-6, thereby inhibiting LPS-induced inflammation in rats (Ávila et al., 2020). Furthermore, a comparative study highlighted the superior inhibitory effect of black goji berry extract on Cyclooxygenase-2 (COX-2) gene expression when compared to red goji berry extract (Magalhães et al., 2022). These findings are attributed to the high concentration of flavonoids and phenolic compounds in wolfberry extracts, which are believed to be responsible for their anti-inflammatory and antioxidant properties (Magiera and Zaręba, 2015; Protti et al., 2017; Lu Y. et al., 2019). For instance, wolfberry flavonoid extract demonstrated antioxidant effects comparable to vitamin C and exhibited anti-inflammatory activity by curbing the production of nitric oxide and pro-inflammatory factors (Yang et al., 2022). Additionally, *L. ruthenicum* extracts exhibited anti-inflammatory effects akin to those of tea polyphenols and reduced oxidative stress (Bi et al., 2023). A positive correlation has also been established between phenolic content and the antioxidant capacities as measured by 2,2-diphenyl-1-picrylhydrazyl (DPPH), 2,2′-Azinobis- (3-ethylbenzthiazoline-6- sulphonate) (ABTS), and Ferric ion reducing antioxidant power (FRAP) assays (Jiang et al., 2021). In the current study, the analysis of three wolfberries revealed a rich abundance of phenolics and flavonoids, suggesting that the anti-inflammatory and antioxidant effects of wolfberries may be attributed to these bioactive components.

Quercetin, isorhamnetin, chrysin, and kaempferol-3-O-rutinoside have been identified as prominent flavonoids in wolfberries (Cid-Ortega and Monroy-Rivera, 2018; Zhang et al., 2019), with quercetin exhibiting the highest relative content among the three wolfberry flavonoids in this study. Quercetin holds significant clinical importance in the treatment of bacterial and viral infections, tumors, diabetes mellitus, hyperlipidemia, and immune system disorders. It is commonly found in the form of glycosides in various plant parts, such as stems, skins, flowers, leaves, buds, seeds, and fruits (Liao et al., 2012). For instance, the extract of *Ceiba pentandra* contains quercetin and rutin, demonstrating anti-tumor effects (Orabi et al., 2024). Similarly, *Equisetum ramosissimum* L. extract, which shares a similar composition with *C. pentandra* extract, has shown efficacy in countering diabetes-induced renal impairment (Abdullah et al., 2024). Additionally, the fruit extract of *Sarcopoterium spinosum*, rich in quercetin, exhibits notable anti-inflammatory and antioxidant properties (Zbeeb et al., 2024). Therefore, quercetin was selected as the primary compound for this study to investigate it is *in vitro* anti-inflammatory effects. The findings revealed that quercetin can exert anti-inflammatory effects by inhibiting the secretion of MCP-1, IL-1β, IFN-*γ*, and reducing the expression of vascular inflammation-producing mediators ICAM-1 and VCAM-1. It has been reported that quercetin exerts its anti-inflammatory effects mainly by inhibiting the production of cytokines (Dower et al., 2015; Carullo et al., 2017). It can reportedly reduce *Streptococcus* suis-induced inflammation by inhibiting the activation of p38 mitogen-activated protein kinase, extracellular signal-related kinases (ERK1/2), and nuclear factor kappa B (NF-κB). It can also reduce the production of pro-inflammatory cytokines such as TNF-α, IL-1β, and IL-6 (Li et al., 2019). It has also been reported that the inhibitory effect of quercetin on inflammatory chemokines is due to IFN-γ and TNF-α induction, which occurs through inhibition of signal transducer and activator of transcription 1 (STAT1) (Kang et al., 2014). Tian et al. found that quercetin derivatives exerted anti-inflammatory effects by decreasing the secretion and expression of TNF and MCP-1, and the effects were superior to those of quercetin (Tian et al., 2017). It can be inferred that the anti-inflammatory properties of wolfberry extracts may be attributed to the prevalence of quercetin and that other quercetin-like constituents are more effective than quercetin. Consequently, the next phase of the research will involve conducting compositional analysis of wolfberry extracts and exploring the anti-inflammatory properties of each compound class. This will not only confirm quercetin as the primary component responsible for the anti-inflammatory effects but also elucidate its underlying mechanism. The outcomes of this research will provide a theoretical foundation for enhancing the added value of wolfberries.

The experimental findings presented in Table 1 have illuminated notable disparities in the relative concentrations of various compounds among the three wolfberries. Specifically, *L. barbarum* exhibited significantly elevated levels of Daminozide, acetaminophen glucuronide, LysoPA 16:0, Catechin, 2-Hydroxyethylphosphonic acid, and Glu-[lycibarbarspermidine F]isomer 3, suggesting these compounds as potential biomarkers for *L. barbarum* identification. Similarly, *L. barbarum* var. *auranticarpum* displayed significantly higher levels of Flavin Single Nucleotide (FMN), 5-Hydroxy-3,7,3′,4′-tetramethoxyflavone (Retusin), Maltol, Hes-peretin-7-*O*-(6″-malonyl)glucoside, and 2-Methyl-3-oxoadipic acid, indicating their utility as distinctive markers for this variant. Furthermore, *L. ruthenicum* presented significantly increased levels of fifteen compounds, including Isoorientin-7-*O*-(6″-p-coumaroyl)glucoside, 4-Methyl-5-thiazoleethanol, Jaceosidin-7-*O*-glucoside, Dihy-dromyricetin-3-*O*-glucoside, Myricetin, Troxerutin, Eriodictyol-8-*C*-glucoside, Hesperetin-7-*O*-rutinoside (Hesperidin), Hesperetin-7-*O*-neohesperidoside (Neohesperidin), Isohemiphloin, 1,8-dihydroxy-2,6-dimethylxanthen-9-one, 3-Hydroxy-3-methylpentane-1,5-dioic acid, 6-*C*-glucosyl-2-hydroxynaringenin, Chrysoe-riol-6,8-di-*C*-glucoside-7-*O*-glucoside, and Quercetin-3-*O*-rutinoside-7-*O*-rhamnoside, which collectively serve as indicative markers for *L. ruthenicum*. These metabolites collectively contribute to the differentiation of the three wolfberries and offer valuable insights for establishing quality benchmarks for each variety.

**TABLE 1:** The marker compounds between different wolfberries.

Types of wolfberries	Ionization model	Molecular weight (Da)	Classification of compounds	Compounds
*L. barbarum*	[M + H]^+^	160.0848	Alkaloids	Daminozide
	[M-H]^-^	327.0954	Others	Acetaminophen glucuronide
	[M-H]^-^	410.2433	Lipids	LysoPA 16:0
	[M-H]^-^	458.0849	Flavonoids	Catechin
	[M-H]^-^	126.0082	Organic acids	2-Hydroxyethylphosphonic acid
	[M + H]^+^	957.3954	Alkaloids	Glu-[lycibarbarspermidine F]isomer3
*L*. *barbarum* var. a*uranticarpum*	[M-H]^-^	456.1046	Nucleotides and derivatives	Flavin Single Nucleotide (FMN)
	[M + H]^+^	358.1053	Flavonoids	5-Hydroxy-3,7,3′,4′-tetramethoxyflavone (Retusin)
	[M + H]^+^	126.0317	Others	Maltol
	[M-H]^-^	550.1323	Flavonoids	Hesperetin-7-*O*-(6″-malonyl)glucoside
	[M-H]^-^	174.0528	Organic acids	2-Methyl-3-oxoadipic acid
*L*. *ruthenicum*	[M + H]^+^	756.1902	Flavonoids	Isoorientin-7-*O*-(6″-p-coumaroyl)glucoside
	[M + H]^+^	143.0405	Others	4-Methyl-5-thiazoleethanol
	[M + H]^+^	492.1268	Flavonoids	Jaceosidin-7-*O*-glucoside
	[M-H]^-^	482.1060	Flavonoids	Dihydromyricetin-3-*O*-glucoside
	[M + H]^+^	318.0376	Flavonoids	Myricetin
	[M + H]^+^	742.2320	Flavonoids	Troxerutin
	[M-H]^-^	450.1162	Flavonoids	Eriodictyol-8-*C*-glucoside
	[M + H]^+^	610.1898	Flavonoids	Hesperetin-7-*O*-rutinoside (Hesperidin)
	[M-H]^-^	610.1898	Flavonoids	Hesperetin-7-*O*-neohesperidoside (Neohesperidin)
	[M-H]^-^	434.1213	Flavonoids	Isohemiphloin
	[M-H]^-^	256.0736	Flavonoids	1,8-dihydroxy-2,6-dimethylxanthen-9-one
	[M-H]^-^	162.0528	Amino acids and derivatives	3-Hydroxy-3-methylpentane-1,5-dioic acid
	[M-H]^-^	450.1162	Flavonoids	6-*C*-glucosyl-2-hydroxynaringenin
	[M + H]^+^	786.2219	Flavonoids	Chrysoeriol-6,8-di-*C*-glucoside-7-*O*-glucoside
	[M + H]^+^	756.2113	Flavonoids	Quercetin-3-*O*-rutinoside-7-*O*-rhamnoside

## 5 Conclusion

In this scholarly investigation, an extensive metabolomic profiling was conducted on three distinct wolfberries, yielding a comprehensive analysis of 1,104 metabolites, which encompassed 211 flavonoids, 176 phenolic acids, and 158 alkaloids. From this extensive dataset, 26 metabolites were identified as potential biomarkers for assessing the quality of wolfberries. Notably, *L*. *ruthenicum* emerged as the superior variety, boasting the highest concentrations of antioxidant compounds and the lowest levels of lipids, thereby positioning it as an optimal selection for health-conscious consumers seeking both antioxidant benefits and low-fat dietary options. The *L*. *barbarum* var. a*uranticarpum*, on the other hand, demonstrated a rich composition of phenolic acids, flavonoids, amino acids, organic acids, and nucleotides such as adenosine 5-phosphate and guanosine 5-phosphate, rendering it an excellent choice for the production of wolfberry-based juices and fresh food products. Furthermore, *in vitro* studies utilizing an LPS-induced HUVECs model indicated that pretreatment with quercetin significantly mitigated inflammatory responses by suppressing the synthesis of inflammatory factors and mediators. These findings suggest that the varying nutritional attributes of the three wolfberries may be linked to their distinct quercetin contents and resultant antioxidant and anti-inflammatory properties. This research furnishes a robust theoretical framework and empirical evidence to support the utilization of wolfberries in both the food and pharmaceutical sectors, thereby advancing the development of wolfberry-derived nutritional and medicinal components. Nonetheless, additional rigorous testing is imperative to fully ascertain the medicinal worth and therapeutic potential of these wolfberries.

## Data Availability

The raw data supporting the conclusion of this article will be made available by the authors, without undue reservation.

## References

[B1] Abdullah R. K. Issa R. A. Abu-Samak M. S. Mohammad B. A. Abbas M. A. Awwad S. H. (2024). Nephroprotective effects of *Equisetum ramosissimum* L. extract in streptozotocin-induced diabetic rats. Pharmacia 71 (8), 1–11. 10.3897/pharmacia.71.e113659

[B2] Abushady E. A. Elagaty S. M. Nassef N. A. Abdelhamid G. S. (2020). The potential hepatoprotective effect of quercetin on cholestatic liver injury in rats. QJM Int. J. Med. 113 (1), i225. 10.1093/qjmed/hcaa065.002

[B3] Ávila C. N. Ribeiro Trindade F. M. Penteado J. O. Janke F. Schneider J. P. Uecker J. N. (2020). Anti-inflammatory effect of a goji berry extract (*Lycium barbarum*) in rats subjected to inflammation by lipopolysaccharides (LPS). Braz. Arch. Biol. Technol. 63, e20180612. 10.1590/1678-4324-2020180612

[B4] Bi Y. Liu X. Liu Y. Wang M. Shan Y. Yin Y. (2023). Molecular and biochemical investigations of the anti-fatigue effects of tea polyphenols and fruit extracts of *Lycium ruthenicum* Murr. on mice with exercise-induced fatigue. Front. Mol. Biosci. 10, 1223411. 10.3389/fmolb.2023.1223411 37416624 PMC10319583

[B5] Carullo G. Cappello A. R. Frattaruolo L. Badolato M. Armentano B. Aiello F. (2017). Quercetin and derivatives: useful tools in inflammation and pain management. Future Med. Chem. 9, 79–93. 10.4155/fmc-2016-0186 27995808

[B6] Ceccherini E. Gisone I. Persiani E. Ippolito C. Falleni A. Cecchettini A. (2024). Novel *in vitro* evidence on the beneficial effect of quercetin treatment in vascular calcification. Front. Pharmacol. 15, 1330374. 10.3389/fphar.2024.1330374 38344172 PMC10853431

[B7] Chen Q. N. Fan J. Q. Lin L. Z. Zhao M. M. (2023). Combination of *Lycium barbarum* L. and Laminaria japonica polysaccharides as a highly efficient prebiotic: optimal screening and complementary regulation of gut probiotics and their metabolites. Int. J. Biol. Macromol. 246, 125534. 10.1016/j.ijbiomac.2023.125534 37355074

[B8] Chinese Pharmacopoeia Commission (2020). Chinese Pharmacopoeia volume 1. Beijing, China: China Medical Science Press, 128–260.

[B9] Cid-Ortega S. Monroy-Rivera J. A. (2018). Extraction of kaempferol and its glycosides using supercritical fluids from plant sources: a review. Food Technol. Biotechnol. 56, 480–493. 10.17113/ftb.56.04.18.5870 30923445 PMC6399721

[B10] Dower J. I. Geleijnse J. M. Gijsbers L. Schalkwijk C. Kromhout D. Hollman P. C. (2015). Supplementation of the pure flavonoids epicatechin and quercetin affects some biomarkers of endothelial dysfunction and inflammation in (Pre)Hypertensive adults: a randomized double-blind, placebo-controlled, crossover trial. J. Nutr. 145, 1459–1463. 10.3945/jn.115.211888 25972527

[B11] Du Y. W. Ma H. Y. Liu Y. Y. Gong R. Lan Y. Zhao J. H. (2024). Major quality regulation network of flavonoid synthesis governing the bioactivity of black wolfberry. New Phytol. 23, 558–575. 10.1111/nph.19602 38396374

[B12] Duan W. H. Zhou L. X. Ren Y. L. Liu F. Xue Y. Z. Wang F. Z. (2024). Lactic acid fermentation of goji berries (*Lycium barbarum*) prevents acute alcohol liver injury and modulates gut microbiota and metabolites in mice. Food & Funct. 15 (3), 1612–1626. 10.1039/d3fo03324d 38240339

[B13] Fakhfakh J. Affes M. Jabeur H. Ayadi M. Allouche N. (2022). The sweet and embellishing *Lycium barbarum* Schweinf. ex Boiss. fruit oil: a potential source of essential ω-6 and ω-9 fatty acids, phytosterols, and carotenoids. Turk J. Chem. 6, 1883–1896. 10.55730/1300-0527.3488 PMC10446916 37621356

[B14] Geng L. L. Liu Z. P. Zhang W. Q. Li W. Wu Z. M. Wang W. (2019). Chemical screen identifies a geroprotective role of quercetin in premature aging. Protein Cell 10 (6), 417–435. 10.1007/s13238-018-0567-y 30069858 PMC6538594

[B15] Gong J. Shen H. Zheng J. Tao N. Gu S. Huang Y. (2016). Identification of key umami-related compounds in Yangtze Coilia ectenes by combining electronic tongue analysis with sensory evaluation. RSC Adv. 6, 45689–45695. 10.1039/c6ra02931k

[B16] Han X. J. Xu T. S. Fang Q. J. Zhang H. J. Yue L. J. Hu G. (2021). Quercetin hinders microglial activation to alleviate neurotoxicity via the interplay between NLRP3 inflammasome and mitophagy. Redox Biol. 44, 102010. 10.1016/j.redox.2021.102010 34082381 PMC8182123

[B17] Heinrich M. Jalil B. Abdel-Tawab M. Echeverria J. Kulić Ž. McGaw L. J. (2022). Best Practice in the chemical characterisation of extracts used in pharmacological and toxicological research-The ConPhyMP-Guidelines. Front. Pharmacol. 13, 953205. 10.3389/fphar.2022.953205 36176427 PMC9514875

[B18] Huang Z. F. Ye Y. L. Long Z. Y. Qin H. H. Liu L. H. Xu A. L. (2023). *Lycium barbarum* polysaccharides improve lipid metabolism disorders of spotted sea bass Lateolabrax maculatus induced by high lipid diet. Int. J. Biol. Macromol. 242 (Pt 3), 125122. 10.1016/j.ijbiomac.2023.125122 37263324

[B19] Islam T. Yu X. M. Badwal T. S. Xu B. J. (2017). Comparative studies on phenolic profiles, antioxidant capacities and carotenoid contents of red goji berry (*Lycium barbarum*) and black goji berry (*Lycium ruthenicum*). Chem. Cent. J. 11 (1), 59. 10.1186/s13065-017-0287-z 29086843 PMC5483215

[B20] Jiang Y. J. Fang Z. X. Leonard W. Zhang P. Z. (2021). Phenolic compounds in *Lycium* berry: composition, health benefits and industrial applications. J. Funct. Foods 77, 104340. 10.1016/j.jff.2020.104340

[B21] Kanehisa M. Goto S. (2000). KEGG: kyoto encyclopedia of genes and genomes. Nucleic Acids Res. 28 (1), 27–30. 10.1093/nar/28.1.27 10592173 PMC102409

[B22] Kang G. J. Han S. C. Kang N. J. Koo D. H. Park D. B. Eun S. Y. (2014). Quercetagetin inhibits macrophage-derived chemokine in HaCaT human keratinocytes via the regulation of signal transducer and activator of transcription 1, suppressor of cytokine signalling 1 and transforming growth factor-β1. Br. J. dermatology 171 (3), 512–523. 10.1111/bjd.12938 24602010

[B23] Li G. Shen X. Wei Y. Si X. Deng X. Wang J. (2019). Quercetin reduces Streptococcus suis virulence by inhibiting suilysin activity and inflammation. Int. Immunopharmacol. 69, 71–78. 10.1016/j.intimp.2019.01.017 30682719

[B24] Li H. M. Tao W. H. Xu X. C. Chen G. L. Ma W. P. Jia S. (2023). *Lycium barbarum* polysaccharides alleviate pancreatic *β*-cells apoptosis through the inhibition of IFN*γ* pathway. J. Funct. Foods. 107, 105706. 10.1016/j.jff.2023.105706

[B25] Liang X. J. Wang Y. J. An W. (2017). Progress of research on germplasm resources of yellow fruit *Lycium barbarum* . Zhejiang Agric. Sci. 58 (8), 1376–1378. 10.16178/j.issn.0528-9017.20170826

[B26] Liang X. J. Wang Y. J. Li Y. K. Zeng X. X. Cao Y. L. An W. (2019). Comparative study on main efficacy components in yellow fruit wolfberry. J. Northwest For. Univ. 34 (5), 108–114.

[B27] Liao H. Zhen Y. H. Ding C. Long Y. Y. Feng X. (2012). Bioactivity mechanisms and pharmacokinetics of quercetin. Prog. Mod. Biomed. 12 (16), 3174–3177+3190. 10.13241/j.cnki.pmb.2012.16.012

[B28] Liu X. X. Fan W. Q. Jiao H. H. Gao H. Tang J. N. Zhu J. Z. (2023). Comparative analysis of differentially expressed genes for biosynthesis of active ingredients in fruits of different cultivars of *Lycium barbarum* L. based on transcriptome sequencing. Chin. J. Biotechnol. 39 (7), 3015–3036. 10.13345/j.cjb.220821 37584145

[B29] Lu L. Mi J. Luo Q. Li Y. K. Liang X. J. Yan Y. M. (2019a). Optimization of the extraction process of total flavonoids from *Lycium barbarum* and its *in vitro* antioxidant activity analysis. Food Industry Sci. Technol. 40 (24), 165–171. 10.13386/j.issn1002-0306.2019.24.027

[B30] Lu Y. Guo S. Zhang F. Yan H. Qian D. Wang H. (2019b). Comparison of functional components and antioxidant activity of *Lycium barbarum* L. fruits from different regions in China. Molecules 24, 2228. 10.3390/molecules24122228 31207958 PMC6632000

[B31] Ma R. X. Zhang M. Yang X. H. Guo J. Fan Y. L. (2023). Transcriptome analysis reveals genes related to the synthesis and metabolism of cell wall polysaccharides in goji berry (*Lycium barbarum* L.) from various regions. J. Sci. Food Agric. 103 (14), 7050–7060. 10.1002/jsfa.12791 37340801

[B32] Magalhães V. Silva A. R. Silva B. Zhang X. Dias A. C. P. (2022). Comparative studies on the anti-neuroinflammatory and antioxidant activities of black and red goji berries. Funct. Foods 92, 105038. 10.1016/j.jff.2022.105038

[B33] Magiera S. Zaręba M. (2015). Chromatographic determination of phenolic acids and flavonoids in *Lycium barbarum* L. and evaluation of antioxidant activity. Food Anal. Methods 8, 2665–2674. 10.1007/s12161-015-0166-y

[B34] Manninen H. Rotola-Pukkila M. Aisala H. Hopia A. Laaksonen T. (2018). Free amino acids and 5’-nucleotides in Finnish forest mushrooms. Food Chem. 247, 23–28. 10.1016/j.foodchem.2017.12.014 29277224

[B35] Oh Y. C. Cho W. K. Im G. Y. Jeong Y. H. Hwang Y. H. Liang C. (2012). Anti-inflammatory effect of *Lycium* Fruit water extract in lipopolysaccharide-stimulated RAW 264.7 macrophage cells. Int. Immunopharmacol. 13 (2), 181–189. 10.1016/j.intimp.2012.03.020 22483979

[B36] Orabi M. A. A. Abouelela M. E. Darwish F. M. M. Abdelkader M. S. A. Elsadek B. E. M. Al Awadh A. A. (2024). *Ceiba pentandra* ethyl acetate extract improves doxorubicin antitumor outcomes against chemically induced liver cancer in rat model: a study supported by UHPLC-Q-TOF-MS/MS identification of the bioactive phytomolecules. Front. Pharmacol. 15, 1337910. 10.3389/fphar.2024.1337910 38370475 PMC10871037

[B37] Peng Y. J. Yan Y. M. Wan P. Chen D. Ding Y. Ran L. W. (2019). Gut microbiota modulation and anti-inflammatory properties of anthocyanins from the fruits of *Lycium ruthenicum* Murray in dextran sodium sulfate-induced colitis in mice. Free Radic. Biol. Med. 136, 96–108. 10.1016/j.freeradbiomed.2019.04.005 30959170

[B38] Peng Y. J. Yan Y. M. Wan P. Dong W. Huang K. Y. Ran L. W. (2020). Effects of long-term intake of anthocyanins from *Lycium ruthenicum* Murray on the organism health and gut microbiota *in vivo* . Food Res. Int. Ott. Ont. 130, 108952. 10.1016/j.foodres.2019.108952 32156393

[B39] Protti M. Gualandi I. Mandrioli R. Zappoli S. Tonelli D. Mercolini L. (2017). Analytical profiling of selected antioxidants and total antioxidant capacity of goji (*Lycium* spp.) berries. J. Pharm. Biomed. 143, 252–260. 10.1016/j.jpba.2017.05.048 28618341

[B40] Song M. Y. Jung H. W. Kang S. Y. Kim K. H. Park Y. K. (2014). Anti-inflammatory effect of *Lycii radicis* in LPS-stimulated RAW 264.7 macrophages. Am. J. Chin. Med. 42 (4), 891–904. 10.1142/S0192415X14500566 25004881

[B41] Song R. L. Wang Z. W. Zhang H. F. (2021). Comparison of nutritional and active ingredients between black wolfberry and red wolfberry. Gourmet Res. 38 (1), 84–87. 10.19913/j.cnki.2095-8730msyj.2021.0011

[B42] Tian H. Liu Q. C. Qin S. C. Zong C. L. Zhang Y. Yao S. T. (2017). Synthesis and cardiovascular protective effects of quercetin 7-O-sialic acid. J. Cell. Mol. Med. 21 (1), 107–120. 10.1111/jcmm.12943 27511707 PMC5192943

[B43] Wang J. Gao H. Y. Xie Y. Wang P. Li Y. Zhao J. L. (2023). *Lycium barbarum* polysaccharide alleviates dextran sodium sulfate-induced inflammatory bowel disease by regulating M1/M2 macrophage polarization via the STAT1 and STAT6 pathways. Front. Pharmacol. 14, 1044576. 10.3389/fphar.2023.1044576 37144216 PMC10151498

[B44] Wang Y. J. Guo S. J. An W. Liu L. Y. Yin Y. Zhang X. Y. (2016). Fruit traits and major nutrients of five species of *Lycium barbarum* . J. For. Environ. 36 (3), 367–372. 10.13324/j.cnki.jfcf.2016.03.019

[B45] Xie Z. Y. Luo Y. Zhang C. J. An W. Zhou J. Jin C. (2023). Integrated metabolome and transcriptome during fruit development reveal metabolic differences and molecular basis between *Lycium barbarum* and *Lycium ruthenicum* . Metabolites 13 (6), 680. 10.3390/metabo13060680 37367839 PMC10303592

[B46] Yang T. Hu Y. Yan Y. Zhou W. Chen G. Zeng X. (2022). Characterization and evaluation of antioxidant and anti-inflammatory activities of flavonoids from the fruits of *Lycium barbarum* . Foods 11, 306. 10.3390/foods11030306 35159457 PMC8834156

[B47] Yang Y. Yu L. Zhu T. Y. Xu S. W. He J. Mao N. N. (2023). Neuroprotective effects of *Lycium barbarum* polysaccharide on light-induced oxidative stress and mitochondrial damage via the Nrf2/HO-1 pathway in mouse hippocampal neurons. Int. J. Biol. Macromol. 251, 126315. 10.1016/j.ijbiomac.2023.126315 37582438

[B48] Zbeeb H. Baldini F. Zeaiter L. Vergani L. (2024). The anti-inflammatory potential of an ethanolic extract from *Sarcopoterium spinosum* fruits for protection and/or counteraction against oxidative stress in dysfunctional endothelial cells. Int. J. Mol. Sci. 25 (3), 1601. 10.3390/ijms25031601 38338880 PMC10855414

[B49] Zhang D. F. Xia T. Dang S. F. Fan G. F. Wang Z. L. (2018). Investigation of Chinese wolfberry (*Lycium spp*.) germplasm by restriction site-associated DNA sequencing (RAD-seq). Biochem. Genet. 56 (6), 575–585. 10.1007/s10528-018-9861-x 29876687 PMC6223726

[B50] Zhang X. Han L. J. Hou S. Z. Raza S. H. A. Wang Z. Y. Yang B. C. (2022). Effects of different feeding regimes on muscle metabolism and its association with meat quality of Tibetan sheep. Food Chem. 374, 131611. 10.1016/j.foodchem.2021.131611 34863603

[B51] Zhang Y. Wang B. Jia Z. Scarlett C. J. Sheng Z. (2019). Adsorption/desorption characteristics and enrichment of quercetin, luteolin and apigenin from *Flos populi* using macroporous resin. Rev. Bras. Farm. 29, 69–76. 10.1016/j.bjp.2018.09.002

[B52] Zheng H. L. Liang X. F. Zhou H. L. Zhou T. Liu X. H. Duan J. L. (2023). Integrated gut microbiota and fecal metabolome analyses of the effect of *Lycium barbarum* polysaccharide on D-galactose-induced premature ovarian insufficiency. Food Funct. 14 (15), 7209–7221. 10.1039/d3fo01659e 37463025

[B53] Zhou L. Wang P. (2018). “Li shizhen's compendium of Materia Medica and poetry culture,” in Proceedings of the 500th anniversary of Li shizhen's birth and 2018 Li shizhen international summit forum on traditional Chinese medicine and great health, 176–181.

[B54] Zhou Q. L. Gong L. H. Ji F. D. Cui Y. J. Mu X. T. Xu H. G. (2021). Comparison of nutrient composition of red wolfberry, yellow wolfberry and black wolfberry. China Brew. 40 (10), 43–49.

